# Genome-wide gene expression changes in postpartum depression point towards an altered immune landscape

**DOI:** 10.1038/s41398-021-01270-5

**Published:** 2021-03-04

**Authors:** Divya Mehta, Karen Grewen, Brenda Pearson, Shivangi Wani, Leanne Wallace, Anjali K. Henders, Elisabeth B. Binder, Vibe G. Frokjaer, Samantha Meltzer-Brody, Naomi R. Wray, Alison M. Stuebe

**Affiliations:** 1 Queensland University of Technology (QUT), Centre for Genomics and Personalised Health, Faculty of Health, Institute of Health and Biomedical Innovation, Kelvin Grove, QLD 4059 Australia; 2 Department of Psychiatry, University of North Carolina School of Medicine, Chapel Hill, NC 27514 USA; 3 Center for Neurostatistics and Statistical Genomics, Institute for Molecular Bioscience, The University of Queensland, St Lucia, QLD 4072 Australia; 4 Max Planck Institute of Psychiatry, Munich, 80804 Germany; 5 Emory University, Atlanta, GA 30322 USA; 6 Neurobiology Research Unit, Copenhagen University Hospital Rigshospitalet, Copenhagen, 2100 Denmark; 7 Center for Integrated Molecular Brain Imaging, Copenhagen University Hospital Rigshospitalet, Copenhagen, 2100 Denmark; 8 Mental Health Services Copenhagen, Copenhagen University Hospital Rigshospitalet, Copenhagen, 2100 Denmark; 9 Division of Maternal-Fetal Medicine, University of North Carolina School of Medicine, Chapel Hill, NC 27514 USA; 10 Department of Obstetrics and Gynecology, School of Medicine, University of North Carolina at Chapel Hill, Chapel Hill, NC 27514 USA; 11 Department of Maternal and Child Health, Carolina Global Breastfeeding Institute, Gillings School of Global Public Health, University of North Carolina at Chapel Hill, Chapel Hill, NC 27514 USA

**Keywords:** Depression, Comparative genomics

## Abstract

Maternal postpartum depression (PPD) is a significant public health concern due to the severe negative impact on maternal and child health and well-being. In this study, we aimed to identify genes associated with PPD. To do this, we investigated genome-wide gene expression profiles of pregnant women during their third trimester of pregnancy and tested the association of gene expression with perinatal depressive symptoms. A total of 137 women from a cohort from the University of North Carolina, USA were assessed. The main phenotypes analysed were Edinburgh Postnatal Depression Scale (EPDS) scores at 2 months postpartum and PPD (binary yes/no) based on an EPDS cutoff of 10. Illumina NextSeq500/550 transcriptomic sequencing from whole blood was analysed using the edgeR package. We identified 71 genes significantly associated with postpartum depression scores at 2 months, after correction for multiple testing at 5% FDR. These included several interesting candidates including TNFRSF17, previously reported to be significantly upregulated in women with PPD and MMP8, a matrix metalloproteinase gene, associated with depression in a genome-wide association study. Functional annotation of differentially expressed genes revealed an enrichment of immune response-related biological processes. Additional analysis of genes associated with changes in depressive symptoms from recruitment to 2 months postpartum identified 66 genes significant at an FDR of 5%. Of these genes, 33 genes were also associated with depressive symptoms at 2 months postpartum. Comparing the results with previous studies, we observed that 15.4% of genes associated with PPD in this study overlapped with 700 core maternal genes that showed significant gene expression changes across multiple brain regions (*P* = 7.9e-05) and 29–53% of the genes were also associated with estradiol changes in a pharmacological model of depression (*P* values range = 1.2e-4–2.1e-14). In conclusion, we identified novel genes and validated genes previously associated with oestrogen sensitivity in PPD. These results point towards the role of an altered immune transcriptomic landscape as a vulnerability factor for PPD.

## Introduction

The perinatal period spanning across pregnancy and postpartum is a vulnerable time for increased mental health issues, which presents several challenges for women and families^1^. Postpartum depression (PPD) is a common mood disorder that occurs in 10–15% of pregnant women^2^. As per the Diagnostic and Statistical Manual (DSM-5, fifth edition), PPD is defined as a major depressive episode that occurs within 4 weeks post-delivery^3^, however many studies investigate depressive symptoms up to 1 year postpartum^4,5^.

Children who are born to mothers with PPD have poor health outcomes compared to the general population including shorter gestation periods, impaired cardiovascular functioning, increased gastrointestinal infections, reduced weight gain and lower respiratory tract infections^6–8^. PPD is also associated with a range of cascading long-term negative health outcomes for the mother and infant including an increased risk of psychiatric disorders and neurodevelopmental deficits such as behavioural problems and learning difficulties in the offspring^9–11^ and perturbed immune system in mothers and maternal suicide^12^. In addition, partners of women with PPD are also at risk of having higher rates of depression than expected^13^.

Despite the high prevalence and negative consequences of PPD, very little is currently known about its underlying biology. Several studies suggest that screening early on in pregnancy can reduce anxiety or depressive symptoms; however, there is very little research and intervention for primary prevention^14,15^. Potential causes for PPD include psychosocial factors such as a history of depression or a psychiatric disorder during pregnancy, inadequate social support, emotional isolation, financial strain, stressful life events and biological factors such as genetic risk and sensitivity to hormonal changes have been proposed^16–18^. Studies have reported the association between single nucleotide polymorphisms in genes including the serotonin transporter-linked promoter region polymorphism (5-HTTLPR)^19^, catechol-O-methyl transferase (COMT), monoamine oxidase (MAO)^20^ and brain-derived neurotrophic factor (BDNF)^21^. However, the pathogenesis of PPD still remains largely obscure.

One potential mechanism for biological changes associated with PPD are changing in the gene expression and/or DNA methylation of certain genes across pregnancy and postpartum. We and others have previously identified a panel of predictive gene expression markers whose transcriptome profiles in the third trimester of pregnancy could predict, with 88% accuracy (within sample), which women were at a higher risk of PPD; the panel was also replicated in an independent sample^22^. In the same study, we further investigated the functional relevance of genes associated with PPD and identified a role for the oestrogen signalling pathway in PPD. In a separate study, we confirmed that the predictive gene expression panel for PPD was also associated with transcriptomic and epigenetic sensitivity to sex-steroid manipulation and mapped onto depressive symptoms and serotonin transporter binding in the brain^23^. Additionally, we have also previously demonstrated that there is an altered peripheral blood transcriptomic landscape in a pharmacological model of sex-steroid induced depression^24^.

To the best of our knowledge, there is no large study that has investigated genome-wide gene expression profiles in PPD using the latest sequencing technologies and there is still a gap in our knowledge of the understanding of the biological mechanisms underlying PPD.

The aim of this study was to identify genes and pathways associated with PPD by investigating global changes in peripheral blood gene expression in association with depressive symptoms in a large cohort of pregnant women. We also aimed to evaluate and cross-validate earlier observations in independent clinical cohorts and test if patterns translate to (estradiol) sex hormone manipulation model.

## Methods

### Participants

Women who were pregnant and had a previous history of a clinical diagnosis of depression were recruited to this study as part of the Mood, Mother and Infant (MMI) study (R01HD073220, mmi.web.unc.edu), an ongoing longitudinal cohort study of mother–infant dyads who were extensively phenotyped during the first postpartum year^25^. The total cohort comprised of 222 mother–infant dyads enrolled in the MMI study. Of these, 164 women drawn from a longitudinal cohort of mother–infant dyads were followed from the third trimester through 12 months postpartum. From these, we selected women who agreed to participate in the genetic component of the study and provided blood sample (*n* = 159) and those with good RNA quality with RNA integrity numbers of over 8, amounting to a total of 137 women. These were the women included in this study. Participants underwent a baseline visit with a structured clinical interview in the third trimester of pregnancy and also postpartum via trained clinicians. The study was approved by the Human Research Ethics Committees of the University of North Carolina. All subjects gave written informed consent to participation. To reduce technical variation, all the patients were recruited from one site, and all had blood collected with the same protocol using the same tubes.

### Clinical measurements

Maternal depressive symptoms during pregnancy and postnatal depressive symptoms were the main outcomes, measured using the Edinburgh Postnatal Depression Scale (EPDS), which has been validated in the antenatal and postnatal periods^26,27^. The EPDS has been recommended as a screening tool for perinatal depression among women worldwide.

Data on postpartum-depressive symptoms were collected 2 months after birth. The total number of postpartum-depressive symptoms was also tallied to obtain a total score (out of 30) and was then coded as a categorical variable (score <10 or score ≥10) to indicate scores suggestive of postpartum-depressive symptoms. The EPDS cut-points used to indicate probable depression were selected based on previously published studies^28,29^. For the study, the EPDS was used as the primary assessment measure and the PPD case definition defined as EPDS score of ≥10 postpartum.

### Gene expression experiments

The total RNA was extracted from whole blood in PAXgene tubes using the PAXgene Blood miRNA Kit from Qiagen. RNA was quantified on the Nanodrop1000 and Qubit™ fluorimeter using the RNA HS Assay Kit followed by a quality check using the Agilent Bioanalyzer RNA 6000 Nano kit. mRNA libraries were prepared using the TruSeq^®^ Stranded mRNA Library Prep Kit for NeoPrep™ following the manufacturer’s instructions. The Qubit Fluorometric quantitation was used to determine the 100 ng input for each sample. The libraries were sequenced as pools of up to 24 libraries. Post library construction each library was quantified using the Qubit™ dsDNA HS Assay Kit, followed by size determination using the Agilent High Sensitivity DNA Kit. Up to 24 unique indexed libraries were equimolar pooled and run on the NextSeq500/550 using NextSeq500/550 High Output v2 kit (75 cycles) following the manufacturer’s instructions at a final concentration of 1.5 pM. Sequencing runs were monitored in real time through Illumina BaseSpace.

RNASeq read filtering, alignment and normalisation were carried out using the RNA Aligner application hosted on BaseSpace. The average percentage of reads passing filter including abundant reads = 21,049,665, with >98% aligned to the human reference genome.

### Statistical analysis

The QC steps were initially performed in R. Raw count data were available from 22,459 genes. The gene-level counts were imported into edgeR and pre-processing involved a filter threshold of >0.7 counts per million (CPM) to remove low expressed genes in at least 50% of the samples followed by trimmed mean of M-values (TMM) normalisation to scale for library size, allowing a total of 12,121 genes for further analysis.

The edgeR package^30^ was used to test for differential expression by fitting a model to the negative binomial distribution with the model including the CPM data against the phenotype of interest (PPD) and adjusting for maternal age, gestational age, current and past use of medication and psychological treatment, BMI and ethnicity. Multiple testing corrections were performed using the false discovery rate (FDR) at 5% threshold.

In silico functional annotation of the differentially expressed genes was performed using the Webgestalt interface^31^. Overrepresentation analysis of the biological processes was done as per the Gene Ontology Functional Database using an FDR for multiple testing correction.

Comparison of differentially expressed genes to previous studies was performed on a gene-level and merging was done using R. First, genes overlapping in the current dataset were merged with those assessed in the previously published study. Next, using R, the two results files from this study and previous study were merged to confirm whether genes differentially expressed in the current study were also significant in that study based on the *P* values of significance. Next, to test whether the overlap of significant genes was more than expected by chance, enrichment testing was performed using 1000 permutations (using random sets) and applying a two-sided Binominal test in R to give a *P* value of enrichment for the comparisons.

## Results

### Demographics

The study comprised 137 women from the MMI study cohort at the University of North Carolina, USA, including 15 women with PPD and 122 with no PPD. Details of the women included in the study are provided in Table 1. All gene expression profiles were measured during recruitment during the third trimester of pregnancy.

**Table 1:** Demographics and clinical characteristics of the samples included in the study.

Phenotype	Mean [SE]/*N* [%]
	Overall sample
Age (in years)	31.39 [0.44]
Gestational age (in weeks)	36.86 [0.12]
Ethnicity—non-Hispanic	110 [86.6%]
BMI pre-pregnancy	26.20 [0.67]
BMI baseline	31.20 [0.62]
Pregnancy number	
1	37 [29.1%]
2	37 [29.1%]
3	28 [22%]
>3	25 [19.8%]
Employment status	
Full-time working	102 [80.3%]
Not working	24 [18.9%]
Other	1 [0.8%]
Highest level of education: high school graduate	10 [7.9%]
Trade or Business college	1 [0.8%]
College	20 [15.7%]
College grad 4th yr	38 [29.9%]
University Postgrad	58 [45.7%]
EPDS baseline	5.20 [0.38]
EPDS 2 months postpartum	4.53 [0.33]
Current smoker	
Current psychotherapy	23 [18.1%]
Current medication	24 [18.9%]
Past psychotherapy	77 [60.6%]
Past medication	70 [55.1%]

### Gene expression associated with postpartum-depressive symptoms

**Fig. 1:**
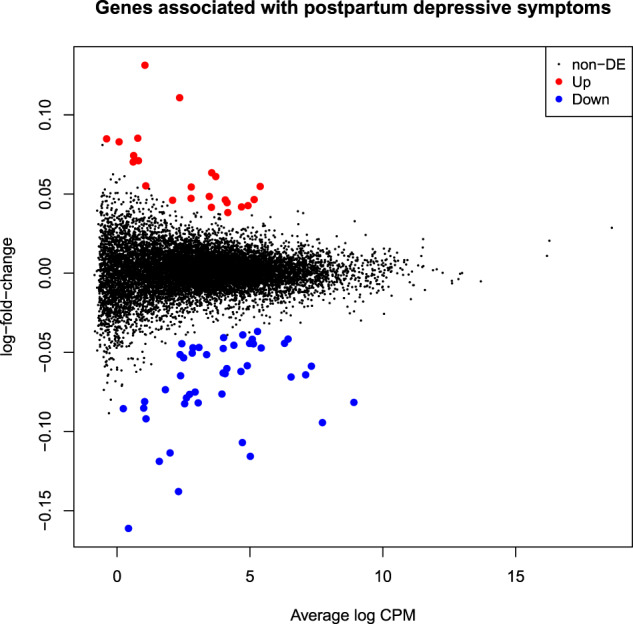
Differentially expressed genes in PPD. MA plot showing up and downregulated genes associated with depressive symptoms at 2 months postpartum.

First, we tested if gene expression profiles of women during the third trimester of pregnancy were significantly associated with depressive symptoms at 2 months postpartum. Through negative binomial regression models using the edgeR package in R^30^, the gene expression profiles were regressed against the quantitative EPDS scores, with maternal age, ethnicity, gestational age in weeks, current and past medications and psychological treatments and BMI included as covariates. In the MMI cohort, a total of 71 genes were significantly associated with depression scores at 2 months postpartum, after correction for multiple testing at 5% FDR (Fig. 1). Among the top genes were *TNFRSR17*, *GYPA*, *JCHAIN*, *SMIM1*, *TRPM8*, *SPATA17*, *HEMGN*, *PRRT4* and *ADAMS15* (Table 2).

**Table 2:** List of differentially expressed genes at the third trimester of pregnancy associated with depressive symptoms at 2 months postpartum at 5% FDR.

Symbol	GnRHa_estradiol	Probe	Chr	*P* value_EPDSpp	FDR_EPDSpp	logFC_diffEPDS	*P* value_diffEPDS	FDR_diffEPDS	logFC_PPD	*P* value_PPD	FDR_PPD	logFC_Dep	*P* value_Dep	FDR_Dep
HBZ	x	3050	16	1.81E-13	7.73E-10	0.08198997	3.96E-11	8.47E-08	1.240623973	8.58E-19	2.75E-15	−0.8915749	1.76E-05	0.02832105
GYPA		2993	4	3.65E-05	0.010910599	0.023796545	0.104618903	0.86184816	0.304788526	0.02543134	0.99980319	−0.0668562	0.73764206	0.99997367
JCHAIN		3512	4	3.02E-07	0.000204157	0.006836049	0.606968986	0.999245113	0.277591843	0.03477346	0.99980319	−0.1611497	0.41273937	0.99997367
SMIM1		388588	1	1.08E-05	0.004463077	−0.046924669	0.000526408	0.081415689	0.522340142	0.00011953	0.04456932	−0.2791593	0.15879646	0.99997367
LOC153910		153910	NA	7.17E-08	7.08E-05	0.038517757	0.037839691	0.647066685	0.017750207	0.92289001	0.99980319	−0.0583912	0.80144348	0.99997367
TRPM8		79054	2	2.95E-05	0.009184138	0.01408958	0.386065356	0.999245113	0.276389445	0.10047632	0.99980319	−0.0771887	0.72789056	0.99997367
TNFRSF17		608	16	2.87E-05	0.009184138	0.023785396	0.126783514	0.901943031	0.377871402	0.01599402	0.95884504	−0.2241632	0.28855385	0.99997367
SPATA17		128153	1	0.000145236	0.032868929	−0.015977987	0.205361204	0.961442886	0.146092805	0.24794379	0.99980319	0.0772507	0.68551058	0.99997367
HLA-DQA2		3118	6	9.23E-06	0.00394781	−0.03402622	0.004663188	0.273869178	−0.23589098	0.07684565	0.99980319	0.39120209	0.05814488	0.99997367
NA		54094	NA	6.80E-08	7.08E-05	0.107471759	3.55E-09	4.55E-06	−0.077278025	0.65099901	0.99980319	0.11434726	0.6089463	0.99997367
HEMGN		55363	9	1.16E-06	0.000678668	0.009033051	0.498373154	0.999245113	0.081613389	0.52344137	0.99980319	0.12426508	0.52030819	0.99997367
PRRT4		401399	7	7.30E-06	0.003231945	−0.019808695	0.224306613	0.965527011	−0.268941122	0.09621023	0.99980319	0.29661201	0.16607037	0.99997367
TMEM158	x	25907	3	0.000220881	0.044129491	0.004957364	0.718388328	0.999245113	−0.08837544	0.50416126	0.99980319	0.26662194	0.17104819	0.99997367
PF4V1		5197	4	0.000244208	0.046140633	−0.012353244	0.364124795	0.999245113	−0.091543585	0.49948912	0.99980319	0.16507481	0.40091434	0.99997367
DIAPH3		81624	13	0.000196028	0.039943103	0.000619347	0.970040483	0.999245113	0.109238181	0.4880814	0.99980319	−0.0752023	0.72118901	0.99997367
CLRN1-AS1		116933	3	5.26E-05	0.015332746	0.030399958	0.033386246	0.621163618	−0.379805572	0.01376195	0.92011568	0.42482939	0.04285816	0.99997367
GDF15		9518	19	0.000145948	0.032868929	−0.034962226	0.038091465	0.647066685	−0.427801989	0.01955796	0.99980319	0.44876145	0.05279787	0.99997367
ADAMTS15		170689	11	1.38E-07	0.000111272	0.066022647	1.68E-05	0.006532214	0.174319176	0.28210173	0.99980319	−0.1148235	0.59692752	0.99997367
ELK2AP		2003	14	3.58E-06	0.001767033	−0.041376595	0.005329177	0.289875588	0.311367498	0.02115396	0.99980319	−0.2259639	0.25708473	0.99997367
ZNF519	x	162655	18	0.000159288	0.034657239	0.002937227	0.845295248	0.999245113	−0.202383477	0.20580551	0.99980319	0.23272153	0.2727202	0.99997367
AZU1		566	19	6.52E-10	1.20E-06	−0.034626939	0.018511889	0.497148793	0.203930581	0.13679875	0.99980319	−0.0489381	0.8063294	0.99997367
PRTN3		5657	19	3.36E-06	0.001750825	−0.037571179	0.025310749	0.573662105	0.006556881	0.96895741	0.99980319	0.14420354	0.49847204	0.99997367
C4BPA	x	722	1	2.91E-10	6.22E-07	−0.051502467	3.73E-06	0.002174605	−0.640051256	6.58E-06	0.00384058	0.82369396	0.00010817	0.11571456
FAM83A		84985	8	6.56E-06	0.003005253	0.035483408	0.024659247	0.565101277	0.682420779	3.88E-06	0.00237108	−0.4020439	0.04915913	0.99997367
CD177		57126	NA	3.00E-05	0.009184138	−0.014578688	0.203567924	0.961442886	0.229194386	0.07083359	0.99980319	−0.4054746	0.03687446	0.99997367
ELANE		1991	19	1.15E-13	7.39E-10	−0.055711655	0.000193525	0.042832372	0.120832578	0.37480411	0.99980319	0.09448629	0.63103503	0.99997367
SLC14A1		6563	18	1.84E-05	0.006388527	0.012590081	0.353435297	0.999245113	−0.119878519	0.35197831	0.99980319	0.35220808	0.06875925	0.99997367
MFSD9		84804	2	0.000248219	0.046179506	0.033478011	0.019777123	0.512886733	0.338344196	0.01966212	0.99980319	−0.2829192	0.15720327	0.99997367
ATOH8		84913	2	1.26E-07	0.000111272	−0.026585915	0.054860764	0.72205758	0.488108301	0.00120052	0.23350081	−0.6060181	0.0030407	0.99997367
FSTL5		56884	4	8.78E-05	0.023000406	−0.033767859	0.050527822	0.708162007	−0.473378851	0.00970811	0.8009674	0.4768658	0.03873353	0.99997367
LOC100132062		100132062	NA	2.74E-59	3.52E-55	−0.09642385	1.86E-08	1.99E-05	−2.079430726	1.58E-33	1.01E-29	2.03759936	5.40E-19	6.94E-15
ALK		238	2	0.000259845	0.047184832	−0.005844187	0.730382863	0.999245113	−0.223973153	0.19563757	0.99980319	0.19379859	0.38496151	0.99997367
PTPRT		11122	20	7.94E-05	0.021243225	−0.009658525	0.540753469	0.999245113	−0.117452457	0.4719397	0.99980319	0.10753096	0.61900473	0.99997367
DEFA1		1667	8	8.27E-09	9.65E-06	−0.034105055	0.011155124	0.406813419	0.324107256	0.01418311	0.92830067	−0.1781284	0.36501703	0.99997367
TPTE		7179	NA	0.000260974	0.047184832	−0.02190591	0.263909072	0.983409969	−0.555091903	0.0068122	0.66302035	0.52221024	0.0339904	0.99997367
MXRA7		439921	17	1.39E-07	0.000111272	0.026352653	0.027553678	0.59646976	0.332170197	0.01493829	0.92830067	−0.3610542	0.06986465	0.99997367
GPRC5C	x	55890	17	8.18E-09	9.65E-06	−0.074305012	1.07E-05	0.005302851	0.377528437	0.02896948	0.99980319	−0.3655967	0.09786856	0.99997367
COBL		23242	7	1.32E-05	0.004967603	−0.010669626	0.534510566	0.999245113	−0.044677683	0.79962307	0.99980319	0.05461506	0.80848884	0.99997367
OLFM4		10562	13	1.18E-05	0.004714007	−0.09056149	1.85E-12	1.09E-08	−0.047752911	0.71452355	0.99980319	0.03402996	0.86046026	0.99997367
NELL1		4745	11	0.000236768	0.046051276	0.00942249	0.545557875	0.999245113	0.192429941	0.22244205	0.99980319	−0.1997864	0.34726165	0.99997367
CEBPE		1053	14	0.000223449	0.044129491	−0.031019039	0.018250128	0.496108596	0.008593278	0.94802827	0.99980319	−0.0084758	0.96512905	0.99997367
SEC14L3		266629	22	1.24E-09	1.99E-06	0.015828663	0.21812967	0.965527011	0.130489444	0.32437928	0.99980319	0.05236892	0.78723598	0.99997367
CTSG		1511	14	4.15E-12	1.06E-08	−0.047720951	0.002517574	0.198318861	0.256940153	0.08033028	0.99980319	0.00679864	0.97110331	0.99997367
LIPN		643418	10	2.61E-05	0.008594769	0.015875193	0.215011061	0.965527011	−0.104090347	0.43029098	0.99980319	0.1067839	0.57903016	0.99997367
NRN1		51299	6	0.000190372	0.03941628	0.025810875	0.090631657	0.832799778	−0.036423626	0.80920195	0.99980319	0.08824533	0.6667064	0.99997367
GRIN2B		2904	12	3.41E-06	0.001750825	−0.048033232	0.016751319	0.476799743	−0.452027194	0.03771923	0.99980319	0.41741015	0.10248191	0.99997367
MZB1		51237	5	2.17E-07	0.000154727	0.035070019	0.009931335	0.380562826	0.316097491	0.02091221	0.99980319	−0.2712525	0.17173474	0.99997367
USP32P1		162632	17	1.23E-05	0.004803545	−0.02265204	0.11552771	0.880715801	0.249454841	0.10004919	0.99980319	−0.4216523	0.04835357	0.99997367
DEFA1B		728358	8	2.73E-09	3.90E-06	−0.082334647	2.83E-08	2.80E-05	0.535661022	5.17E-05	0.02287903	−0.505308	0.00958881	0.99997367
XKR3		150165	22	3.51E-12	1.06E-08	0.020147807	0.157623182	0.94242466	−0.090729408	0.55190992	0.99980319	0.12220984	0.55636885	0.99997367
SHISA6		388336	17	0.00017958	0.03812032	−0.049507351	0.004773282	0.274774096	−0.057699705	0.74644417	0.99980319	0.10304895	0.64837734	0.99997367
RAB3IL1		5866	11	9.19E-05	0.023607002	−0.002823157	0.833233945	0.999245113	−0.018794745	0.88737441	0.99980319	0.16526773	0.39493394	0.99997367
BPI		671	20	0.000128913	0.031223752	−0.047161513	0.000484145	0.076727959	−0.373528161	0.00363043	0.45246397	0.34982249	0.06941875	0.99997367
OR8U8		504189	NA	7.64E-05	0.020856842	0.042692465	0.000391896	0.065334587	0.516211389	9.78E-05	0.03924713	−0.3413738	0.08193914	0.99997367
TFF3		7033	21	0.000181144	0.03812032	−0.027929543	0.05632791	0.723949792	−0.539616026	0.00039369	0.10313927	0.5280528	0.01157969	0.99997367
CNBD1		168975	8	1.73E-05	0.006183318	0.01670876	0.332781984	0.999245113	0.022683504	0.9025405	0.99980319	0.03581888	0.87235199	0.99997367
PPARGC1A	x	10891	4	7.58E-05	0.020856842	−0.020666423	0.220000414	0.965527011	−0.181135049	0.30375862	0.99980319	0.19450487	0.38919359	0.99997367
CPA5	x	93979	NA	0.000105965	0.026159008	−0.069099835	4.80E-06	0.002567843	−0.119843608	0.44801674	0.99980319	0.19377932	0.35625076	0.99997367
CLEC12A	x	160364	12	0.000137021	0.031980631	−0.046454778	3.37E-05	0.0109922	0.209864679	0.08989106	0.99980319	−0.0452303	0.81056457	0.99997367
DEFA4		1669	8	3.58E-07	0.000230067	−0.065234631	3.52E-06	0.002148751	0.161740923	0.22345996	0.99980319	−0.0154213	0.93716872	0.99997367
BCAM		4059	19	2.22E-05	0.007486807	0.018351203	0.158160908	0.94242466	−0.189520866	0.15008212	0.99980319	0.37806366	0.05108819	0.99997367
SHISA7		729956	19	4.27E-06	0.002031316	0.025612122	0.154200044	0.938547656	0.243275156	0.16087353	0.99980319	−0.1664301	0.44848875	0.99997367
PLCB4	x	5332	20	0.000244416	0.046140633	−0.019949231	0.273279271	0.989116508	0.141980609	0.45338217	0.99980319	−0.0934137	0.68937109	0.99997367
LTF		4057	3	0.000136734	0.031980631	−0.058823471	1.34E-05	0.005903106	−0.464815718	0.00024729	0.07054463	0.38819801	0.0434471	0.99997367
RASAL2		9462	1	1.59E-05	0.005845119	−0.096580109	2.55E-12	1.09E-08	−0.496078584	0.00030139	0.08060174	0.63127969	0.00207093	0.99997367
TACSTD2		4070	1	0.000104739	0.026159008	−0.001236843	0.932804732	0.999245113	0.212247984	0.18805412	0.99980319	−0.2836035	0.18378057	0.99997367
PRSS33	x	260429	16	1.82E-06	0.00101726	−0.017543158	0.204453261	0.961442886	0.669401492	7.80E-07	0.00077909	−0.8128711	3.66E-05	0.05215136
AOC1		26	7	2.14E-07	0.000154727	−0.03220397	0.058722646	0.736808196	−0.038105907	0.82650662	0.99980319	−0.1190881	0.58675507	0.99997367
LINC01122		400955	2	0.000151692	0.033573551	−0.010025233	0.562863428	0.999245113	−0.365763605	0.04535562	0.99980319	0.38442049	0.09844397	0.99997367
TRPM3		80036	9	6.48E-05	0.018486308	0.029584207	0.051525843	0.709258838	0.126190993	0.41924413	0.99980319	−0.0668866	0.75091325	0.99997367
TMTC1		83857	12	4.15E-07	0.000253841	0.008189727	0.481246148	0.999245113	0.067323981	0.6281319	0.99980319	−0.3685774	0.06898419	0.99997367

*EPDS* Edinburgh Postnatal Depression Scale symptoms at 2 months postpartum, *diffEPDS* difference in EPDS symptoms from third trimester pregnancy to 2 months postpartum, *PPD* EPDS symptoms in the PPD group, *Dep* EPDS symptoms in the depression group, *GnRHa_estradiol* genes associated with estradiol in the GnRHa study, *FDR* *P* values after false discovery rate multiple testing.

**Fig. 2:**
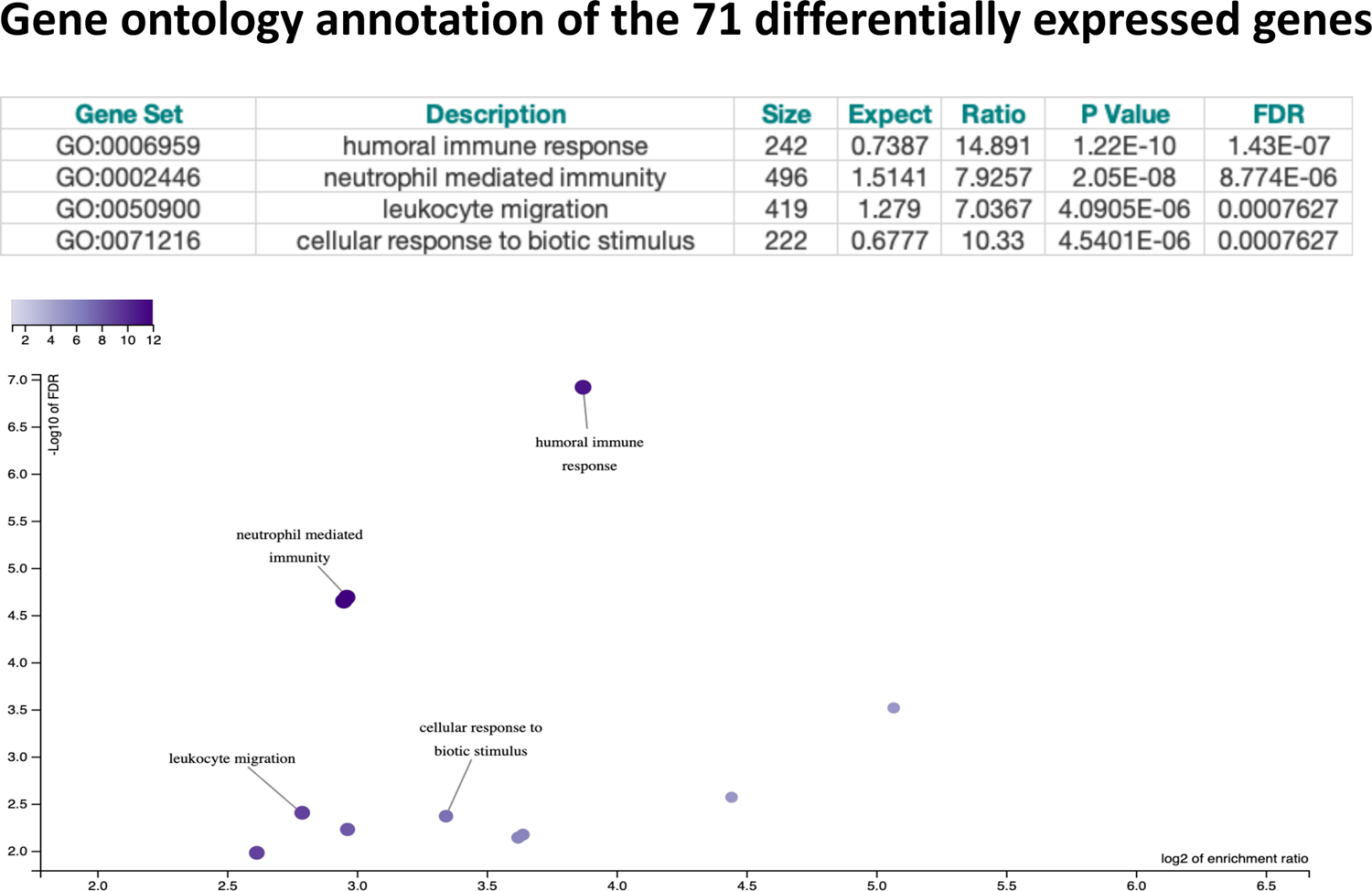
Functional annotation. Functional annotation of the differentially expressed genes associated with depressive symptoms at 2 months postpartum.

Using the Webgestalt interface, we found that the 71 genes were enriched for several immune-related categories as per the Gene Ontology Functional Database (Fig. 2) and the genes were overrepresented within the neutrophil degranulation (*P* = 1.1E-9, *n* = 13 genes), innate immunity (*P* = 1.8E-6, *n* = 14 genes) and fibrin clot formation (*P* = 4.5E-5, *n* = 3 genes) pathways using the Reactome Pathway database.

### Gene expression associated with a change in postpartum-depressive symptoms

The gene expression profiles were regressed against the changes in EPDS scores, with maternal age, ethnicity, gestational age in weeks, current and past medications and psychological treatments and BMI included as covariates. When assessing gene expression profiles significantly associated with changes in depressive symptoms from recruitment to 2 months postpartum (2 months postpartum EPDS–EPDS at recruitment), a total of 66 genes were significant at an FDR of 5% in the MMI cohort.

Of these genes, 33 genes were also significantly associated (*P* < 0.05) with depressive symptoms at 2 months postpartum and of these 14 remained significant at 5% FDR. The genes included *OLFM4, RASAL2, BHZ, DEFA1B, DEFA4, C4BPA, CPA5, GPRC5C, LTF, ADAMTS15, CLEC12A* and *ELANE* (Table 3).

**Table 3:** List of differentially expressed genes at the third trimester of pregnancy associated with changes in depressive symptoms (from the third trimester to 2 months postpartum) at 5% FDR.

Symbol	GnRHa_estradiol	Probe	Chr	*P* value_EPDSv2	FDR_EPDSv2	logFC_diffEPDSv2	*P* value_diffEPDSv2	FDR_diffEPDSv2	logFC_PPD	*P* value_PPD	FDR_PPD	logFC_Dep	*P* value_Dep	FDR_Dep
SLC12A1	x	6557	15	0.738374001	0.999797241	−0.143662302	3.04E-23	3.91E-19	−1.514853079	3.68E-24	1.58E-20	1.52947173	1.01E-10	4.31E-07
OLFM4		10562	13	1.18E-05	0.004714007	−0.09056149	1.85E-12	1.09E-08	−0.047752911	0.714523555	0.99980319	0.03402996	0.86046026	0.99997367
RASAL2		9462	1	1.59E-05	0.005845119	−0.096580109	2.55E-12	1.09E-08	−0.496078584	0.000301385	0.08060174	0.63127969	0.00207093	0.99997367
ZFP57	x	346171	6	0.688934236	0.999797241	0.113752939	9.57E-12	3.07E-08	0.629257704	0.000143791	0.04988752	−0.6042326	0.0056805	0.99997367
MUC6	x	4588	11	0.032615955	0.835444211	0.107037187	2.20E-11	5.65E-08	0.068004697	0.67455073	0.99980319	−0.2445742	0.26286793	0.99997367
HBZ	x	3050	16	1.81E-13	7.73E-10	0.08198997	3.96E-11	8.47E-08	1.240623973	8.58E-19	2.75E-15	−0.8915749	1.76E-05	0.02832105
PNMA3		29944	X	0.588321779	0.999797241	0.084017297	1.38E-09	2.52E-06	−0.048544382	0.737626973	0.99980319	−0.0740358	0.71439951	0.99997367
RAP1GAP		5909	1	0.000417001	0.066086904	−0.084706388	1.69E-09	2.71E-06	−0.700111217	1.09E-07	0.0001401	0.95024605	2.17E-06	0.00537669
IFI27		3429	NA	0.491717731	0.999797241	0.088816879	2.79E-09	3.98E-06	1.079393826	5.46E-16	1.40E-12	−0.7622081	8.16E-05	0.10472842
NA		54094	NA	6.80E-08	7.08E-05	0.107471759	3.55E-09	4.55E-06	−0.077278025	0.650999011	0.99980319	0.11434726	0.6089463	0.99997367
ENC1		8507	5	0.005321833	0.336533844	−0.072184102	1.08E-08	1.26E-05	−0.282262065	0.032073728	0.99980319	0.22215453	0.25746315	0.99997367
LOC100132062		100132062	NA	2.74E-59	3.52E-55	−0.09642385	1.86E-08	1.99E-05	−2.079430726	1.58E-33	1.01E-29	2.03759936	5.40E-19	6.94E-15
DEFA1B		728358	8	2.73E-09	3.90E-06	−0.082334647	2.83E-08	2.80E-05	0.535661022	5.17E-05	0.02287903	−0.505308	0.00958881	0.99997367
SLC35F1		222553	6	0.012780901	0.533803126	−0.070659963	1.20E-07	0.000110253	0.899503922	2.61E-10	4.78E-07	−0.7774074	0.00015966	0.14639261
FAM3B		54097	21	0.000506343	0.073862832	−0.081062951	4.36E-07	0.000357438	−1.206842502	2.86E-11	6.13E-08	1.29211562	4.09E-08	0.00013115
A2M		2	12	0.125486579	0.999797241	0.074797133	4.46E-07	0.000357438	0.066016866	0.674833048	0.99980319	−0.1488668	0.48318013	0.99997367
RNF182		221687	6	0.983664265	0.999797241	−0.08587623	5.84E-07	0.000441361	−0.820514565	1.54E-08	2.47E-05	0.9490668	7.75E-06	0.01421399
CLDND2		125875	19	0.401412478	0.999797241	0.070517883	1.26E-06	0.000899767	0.016645892	0.912012943	0.99980319	−0.1021826	0.6269187	0.99997367
COL5A3	x	50509	19	0.172251596	0.999797241	0.071123394	1.60E-06	0.001082012	0.059865205	0.690177553	0.99980319	−0.2220476	0.27428873	0.99997367
ANKRD36B		57730	2	0.403733504	0.999797241	0.083729733	1.92E-06	0.001230403	−0.148062332	0.425223241	0.99980319	0.19293391	0.40812337	0.99997367
DEFA4		1669	8	3.58E-07	0.000230067	−0.065234631	3.52E-06	0.002148751	0.161740923	0.223459958	0.99980319	−0.0154213	0.93716872	0.99997367
C4BPA	x	722	1	2.91E-10	6.22E-07	−0.051502467	3.73E-06	0.002174605	−0.640051256	6.58E-06	0.00384058	0.82369396	0.00010817	0.11571456
MICU1		10367	10	0.008452083	0.439583873	0.071183702	4.67E-06	0.002567843	−0.393099561	0.01809445	0.99980319	0.4094252	0.05992712	0.99997367
CPA5	x	93979	NA	0.000105965	0.026159008	−0.069099835	4.80E-06	0.002567843	−0.119843608	0.448016742	0.99980319	0.19377932	0.35625076	0.99997367
NT5C3A		51251	7	0.002112895	0.191346638	0.064676607	5.26E-06	0.002701077	0.00076943	0.995962869	0.99980319	0.01731807	0.935702	0.99997367
GPRC5C	x	55890	17	8.18E-09	9.65E-06	−0.074305012	1.07E-05	0.005302851	0.377528437	0.028969482	0.99980319	−0.3655967	0.09786856	0.99997367
LINC00854		100874261	17	0.89885143	0.999797241	0.063317275	1.17E-05	0.005562922	0.107322993	0.468550742	0.99980319	−0.1956506	0.34744068	0.99997367
VNN1		8876	6	0.046944791	0.982428275	−0.049143107	1.27E-05	0.005837152	−0.032198571	0.799444025	0.99980319	0.04643599	0.80871155	0.99997367
LTF		4057	3	0.000136734	0.031980631	−0.058823471	1.34E-05	0.005903106	−0.464815718	0.000247294	0.07054463	0.38819801	0.0434471	0.99997367
FAM153B		202134	5	0.011646873	0.515005387	0.076226915	1.38E-05	0.005903106	−0.354084609	0.063289465	0.99980319	0.35484633	0.13436291	0.99997367
BRSK2		9024	11	0.184299576	0.999797241	0.076659523	1.52E-05	0.006217663	−0.440448095	0.0242044	0.99980319	0.37176057	0.12460352	0.99997367
FAM53A		152877	4	0.798983611	0.999797241	0.075963322	1.55E-05	0.006217663	0.2412146	0.188313624	0.99980319	−0.268703	0.24084011	0.99997367
ADAMTS15		170689	11	1.38E-07	0.000111272	0.066022647	1.68E-05	0.006532214	0.174319176	0.282101731	0.99980319	−0.1148235	0.59692752	0.99997367
COL6A3		1293	2	0.307133445	0.999797241	0.062579979	1.91E-05	0.007027999	0.146175885	0.347679407	0.99980319	−0.0608776	0.77358719	0.99997367
LOC100507424		100507424	NA	0.123121497	0.999797241	0.054632332	1.92E-05	0.007027999	−0.142127217	0.304077778	0.99980319	0.03929275	0.84720725	0.99997367
OBSCN		84033	1	0.722086924	0.999797241	0.050750803	2.28E-05	0.008116835	0.071759756	0.571960158	0.99980319	−0.1721324	0.37273278	0.99997367
HS2ST1		9653	1	0.013911554	0.553838011	0.064543649	2.43E-05	0.008422227	0.06095681	0.703847531	0.99980319	−0.071729	0.73247663	0.99997367
NPR2	x	4882	9	0.313616353	0.999797241	0.07470813	2.91E-05	0.009837403	0.015369885	0.929490553	0.99980319	−0.1003678	0.66059056	0.99997367
CLEC12A	x	160364	12	0.000137021	0.031980631	−0.046454778	3.37E-05	0.0109922	0.209864679	0.089891059	0.99980319	−0.0452303	0.81056457	0.99997367
TMEM91		641649	19	0.404895127	0.999797241	0.058584845	3.43E-05	0.0109922	−0.070196956	0.64309492	0.99980319	−0.0135671	0.94490559	0.99997367
LINC00189		193629	21	0.010618077	0.493855975	0.056343712	3.87E-05	0.012116049	−0.298187073	0.037427634	0.99980319	0.29577551	0.1414945	0.99997367
C4B_2		100293534	NA	0.007119309	0.388896016	0.055595437	3.96E-05	0.012116049	0.495547097	0.000181471	0.05762403	−0.170722	0.37935001	0.99997367
PLCH2		9651	1	0.121882192	0.999797241	0.053982765	5.72E-05	0.017080892	−0.123053219	0.378586968	0.99980319	−0.0175891	0.92956679	0.99997367
KCNMA1		3778	10	0.004948314	0.321229175	0.055323213	6.44E-05	0.018566121	0.115752688	0.43053291	0.99980319	−0.2240934	0.28222647	0.99997367
NPIPB5		100132247	16	0.363761914	0.999797241	0.053370079	6.51E-05	0.018566121	0.233122434	0.094712932	0.99980319	−0.358436	0.07588194	0.99997367
LOC100132077		100132077	NA	0.003168783	0.252656315	0.055771593	8.95E-05	0.024969396	−0.290148219	0.053014626	0.99980319	0.28559265	0.17196614	0.99997367
CLEC12B		387837	12	0.000839057	0.105597818	−0.045399984	0.000104155	0.028447571	−0.125781635	0.339340239	0.99980319	0.28429311	0.14471212	0.99997367
DTX2P1-UPK3BP1-PMS2P11		441263	NA	0.697163808	0.999797241	0.054641303	0.00010951	0.029287154	0.019767196	0.891894338	0.99980319	−0.0486238	0.80942813	0.99997367
PRKXP1		441733	15	0.989146813	0.999797241	0.047857927	0.000122714	0.03195716	0.264657472	0.053607631	0.99980319	−0.3048762	0.12455578	0.99997367
MAML2		84441	11	0.054972619	0.999797241	0.047067694	0.000125186	0.03195716	−0.202750932	0.115280757	0.99980319	0.17016595	0.3787149	0.99997367
DDR1	x	780	6	0.528160627	0.999797241	0.056339661	0.000127967	0.03195716	0.10239741	0.500742883	0.99980319	−0.2130413	0.30183844	0.99997367
CDC42BPG		55561	11	0.740909675	0.999797241	0.055678667	0.000129452	0.03195716	0.144699948	0.344577413	0.99980319	−0.2834911	0.17133707	0.99997367
KIF19		124602	17	0.674283974	0.999797241	0.064295989	0.000149316	0.036165474	−0.31711123	0.086336356	0.99980319	0.36479985	0.11773515	0.99997367
UBAC2	x	337867	13	0.776875995	0.999797241	0.064645112	0.000163489	0.038864885	−0.115184307	0.521453911	0.99980319	0.09990956	0.66283344	0.99997367
FIBCD1		84929	9	0.01477133	0.571957401	0.054000943	0.00016668	0.038903107	0.72292732	2.77E-06	0.00197483	−0.5574507	0.007439	0.99997367
KLRC3	x	3823	12	0.956279673	0.999797241	0.0509244	0.000178701	0.040964013	0.108077118	0.445135655	0.99980319	−0.0246341	0.9013113	0.99997367
RASA2	x	5922	3	0.033272719	0.844114418	0.047235684	0.000183238	0.041267076	−0.267466884	0.043001433	0.99980319	0.26920447	0.16864055	0.99997367
ELANE		1991	19	1.15E-13	7.39E-10	−0.055711655	0.000193525	0.042832372	0.120832578	0.374804112	0.99980319	0.09448629	0.63103503	0.99997367
PVALB		5816	22	0.006261676	0.365368784	−0.057766662	0.000215201	0.045767566	0.141207644	0.392691699	0.99980319	0.01047018	0.9611797	0.99997367
MS4A3	x	932	11	0.000798942	0.10346369	−0.049997885	0.000218028	0.045767566	−0.082803294	0.53179566	0.99980319	0.19154916	0.3263714	0.99997367
SLC26A3		1811	7	0.672369503	0.999797241	0.054041842	0.0002224	0.045767566	0.284708866	0.063130844	0.99980319	−0.1980125	0.34245232	0.99997367
ACHE		43	NA	0.994744858	0.999797241	−0.060368494	0.000222854	0.045767566	−0.310915524	0.053342115	0.99980319	0.46295071	0.03073028	0.99997367
ISL2		64843	15	0.001486002	0.153837193	−0.048382632	0.000224613	0.045767566	−0.273752693	0.049356113	0.99980319	0.25044517	0.21361948	0.99997367
KRT73		319101	12	0.820103696	0.999797241	0.051206891	0.000229713	0.046075372	0.14722346	0.313700428	0.99980319	−0.2500906	0.21348809	0.99997367
ARRDC4		91947	15	0.377622927	0.999797241	−0.045100521	0.00023539	0.046487629	−0.486360588	0.000256439	0.07156327	0.43111988	0.02688404	0.99997367
MYH7B		57644	20	0.703269593	0.999797241	0.049995062	0.000256825	0.049952474	−0.018903827	0.89554412	0.99980319	−0.0695272	0.73062912	0.99997367

*EPDS* Edinburgh Postnatal Depression Scale symptoms at 2 months postpartum, *diffEPDS* difference in EPDS symptoms from the third trimester of pregnancy to 2 months postpartum, *PPD* EPDS symptoms in the PPD group, *Dep* EPDS symptoms in the depression group, *GnRHa_estradiol* genes associated with estradiol in the GnRHa study, *FDR* *P* values after False Discovery rate multiple testing.

### Gene expression associated with postpartum depression onset

Next, we assessed the onset of PPD at 2 months postpartum. First, the women were split into three groups

i. PPD-onset group—these were women with EPDS < 10 at recruitment and EPDS ≥ 10 at postpartum (anytime between 1 and 12 months postpartum) (*N* = 25)
ii. Depressed group—women with ≥ 10 EPDS at recruitment and ≥10 EPDS at postpartum (anytime between 1 and 12 months postpartum) (*N* = 44)
iii. Control/euthymic group—women with <10 EPDS at recruitment and postpartum (anytime between 1 and 12 months postpartum) (*N* = 68).

Separate regressions were performed to compare the PPD and depressed groups to the control groups, to identify PPD-onset specific genes. The gene expression profiles were regressed against the group status, with maternal age, ethnicity, gestational age in weeks, current and past medications and psychological treatments and BMI included as covariates. A total of 38 genes were significantly differentially expressed between the PPD and the control groups (Table 4), and of these, 8 genes overlapped with those differentially expressed between the depressed and control groups, indicating that 30 genes showed PPD-onset specific gene expression. Of the 30 genes, 16 genes were also significant in the difference in depressive symptoms (*P* < 0.05). The full analysis results are shown in Supplementary Table 1.

**Table 4:** List of differentially expressed genes at the third trimester of pregnancy associated with changes in depressive symptoms (from the third trimester to 2 months postpartum) at 5% FDR.

Symbol	GnRHa_estradiol	Probe	Chr	*P* value_EPDSv2	FDR_EPDSv2	logFC_diffEPDSv2	*P* value_diffEPDSv2	FDR_diffEPDSv2	logFC_PPD	*P* value_PPD	FDR_PPD	logFC_Dep	*P* value_Dep	FDR_Dep
LOC100132062		100132062	NA	2.74E-59	3.52E-55	−0.09642385	1.86E-08	1.99E-05	−2.0794307	1.58E-33	1.01E-29	2.03759936	5.40E-19	6.94E-15
GSTA7P		730152	6	0.147252834	0.999797241	0.072537172	0.000661439	0.094343313	2.272172	1.65E-36	2.12E-32	−1.865593	1.46E-14	9.40E-11
SLC12A1	x	6557	15	0.738374001	0.999797241	−0.143662302	3.04E-23	3.91E-19	−1.5148531	3.68E-24	1.58E-20	1.52947173	1.01E-10	4.31E-07
FAM3B		54097	21	0.000506343	0.073862832	−0.081062951	4.36E-07	0.000357438	−1.2068425	2.86E-11	6.13E-08	1.29211562	4.09E-08	0.00013115
RAP1GAP		5909	1	0.000417001	0.066086904	−0.084706388	1.69E-09	2.71E-06	−0.7001112	1.09E-07	0.0001401	0.95024605	2.17E-06	0.00537669
MYOM2		9172	NA	0.558974564	0.999797241	0.045488778	0.002563317	0.19942602	0.66107629	8.04E-07	0.00077909	−0.9456007	2.51E-06	0.00537669
RNF182		221687	6	0.983664265	0.999797241	−0.08587623	5.84E-07	0.000441361	−0.8205146	1.54E-08	2.47E-05	0.9490668	7.75E-06	0.01421399
HBZ		3050	16	1.81E-13	7.73E-10	0.08198997	3.96E-11	8.47E-08	1.24062397	8.58E-19	2.75E-15	−0.8915749	1.76E-05	0.02832105
PRSS33	x	260429	16	1.82E-06	0.00101726	−0.017543158	0.204453261	0.961442886	0.66940149	7.80E-07	0.00077909	−0.8128711	3.66E-05	0.05215136
IFI27		3429	NA	0.491717731	0.999797241	0.088816879	2.79E-09	3.98E-06	1.07939383	5.46E-16	1.40E-12	−0.7622081	8.16E-05	0.10472842
PTGDR2		11251	11	0.07345098	0.999797241	−0.009817193	0.48417276	0.999245113	0.68209765	9.10E-07	0.00077909	−0.7728273	9.83E-05	0.11468724
C4BPA	x	722	1	2.91E-10	6.22E-07	−0.051502467	3.73E-06	0.002174605	−0.6400513	6.58E-06	0.00384058	0.82369396	0.00010817	0.11571456
MAPK8IP1P2		644172	NA	0.038012933	0.90051655	0.000303088	0.987530803	0.999245113	−0.9800435	2.22E-06	0.001679	0.97855722	0.00012366	0.12211055
SLC35F1		222553	6	0.012780901	0.533803126	−0.070659963	1.20E-07	0.000110253	0.89950392	2.61E-10	4.78E-07	−0.7774074	0.00015966	0.14639261
CMBL		134147	5	0.475930622	0.999797241	−0.033792967	0.036959174	0.639413638	−0.7375122	3.29E-06	0.00211048	0.74263329	0.00044747	0.36562804
SFRP2		6423	4	0.422133267	0.999797241	−0.019762377	0.182110005	0.957043264	−0.5742315	2.74E-05	0.01256455	0.70217911	0.00045572	0.36562804
GALNT15		117248	3	0.661378861	0.999797241	0.005687522	0.71076802	0.999245113	0.73127667	5.79E-07	0.00067543	−0.678052	0.00076939	0.57668405
ALOX15		246	17	0.006381561	0.367354704	−0.004218688	0.757440501	0.999245113	0.57396501	1.79E-05	0.00886108	−0.6573506	0.00080862	0.57668405
RAVER2		55225	1	0.122015102	0.999797241	0.026473215	0.082808829	0.811263458	0.73076056	8.57E-07	0.00077909	−0.6602258	0.00122902	0.83036233
HES6		55502	2	0.616984359	0.999797241	−0.023906291	0.104221966	0.860937819	0.78046567	2.83E-08	4.04E-05	−0.5834678	0.00300491	0.99997367
SPTBN4	x	57731	19	0.853952516	0.999797241	0.04814081	0.003906481	0.251997482	0.84111298	1.57E-06	0.00125925	−0.5715084	0.01091626	0.99997367
FIBCD1		84929	9	0.01477133	0.571957401	0.054000943	0.00016668	0.038903107	0.72292732	2.77E-06	0.00197483	−0.5574507	0.007439	0.99997367
OLR1		4973	12	0.137268091	0.999797241	−0.040708626	0.002826989	0.21222253	−0.6437111	3.00E-06	0.00202536	0.62579802	0.00157598	0.99997367
FAM83A		84985	8	6.56E-06	0.003005253	0.035483408	0.024659247	0.565101277	0.68242078	3.88E-06	0.00237108	−0.4020439	0.04915913	0.99997367
RSAD2		91543	2	0.001262375	0.135042605	−0.003101777	0.802973454	0.999245113	0.55847735	1.04E-05	0.00581173	−0.423053	0.02966682	0.99997367
NA		654341	NA	0.040041391	0.918606088	0.043642091	0.002347612	0.194240656	−0.5985625	1.47E-05	0.00779562	0.62713957	0.00188074	0.99997367
DDX11L10		100287029	16	0.027398604	0.771306761	0.019363117	0.196566971	0.961362007	−0.7114069	1.52E-05	0.00779562	0.60633309	0.00556542	0.99997367
NCAPG2		54892	NA	0.118956575	0.999797241	0.035486631	0.013137006	0.436890536	0.60702078	2.18E-05	0.01035033	−0.4730099	0.01883019	0.99997367
DEFA1B		728358	8	2.73E-09	3.90E-06	−0.082334647	2.83E-08	2.80E-05	0.53566102	5.17E-05	0.02287903	−0.505308	0.00958881	0.99997367
MMP8		4317	11	0.093862874	0.999797241	−0.042863638	0.001119109	0.133018019	−0.5140184	6.88E-05	0.02943342	0.50413614	0.00877009	0.99997367
NEBL		10529	10	0.177260886	0.999797241	−0.030514743	0.029510787	0.604610542	0.59512746	7.33E-05	0.03037117	−0.6234225	0.00358599	0.99997367
OR8U8		504189	NA	7.64E-05	0.020856842	0.042692465	0.000391896	0.065334587	0.51621139	9.78E-05	0.03924713	−0.3413738	0.08193914	0.99997367
CLC		1178	19	0.04198233	0.934015898	0.001050171	0.933703936	0.999245113	0.48764167	0.00010431	0.0405769	−0.5586975	0.00285235	0.99997367
SMIM1		388588	1	1.08E-05	0.004463077	−0.046924669	0.000526408	0.081415689	0.52234014	0.00011953	0.04456932	−0.2791593	0.15879646	0.99997367
ISG15		9636	1	0.335644265	0.999797241	0.016722871	0.173604797	0.957043264	0.49763474	0.00012152	0.04456932	−0.3898466	0.04277624	0.99997367
KIAA1324L		222223	7	0.940093172	0.999797241	0.011875341	0.433620456	0.999245113	−0.6272798	0.0001271	0.04532091	0.603388	0.00521569	0.99997367
ZFP57	x	346171	6	0.688934236	0.999797241	0.113752939	9.57E-12	3.07E-08	0.6292577	0.00014379	0.04988752	−0.6042326	0.0056805	0.99997367
TMEM176B	x	28959	7	0.003678342	0.266546287	−9.93E-05	0.993610049	0.999245113	0.48636354	0.00014793	0.04997309	−0.6027717	0.0021531	0.99997367

*EPDS* Edinburgh Postnatal Depression Scale symptoms at 2 months postpartum, *diffEPDS* difference in EPDS symptoms from the third trimester of pregnancy to 2 months postpartum, *PPD* EPDS symptoms in the PPD group, *Dep* EPDS symptoms in the depression group, *GnRHa_estradiol* genes associated with estradiol in the GnRHa study, *FDR* *P* values after false discovery rate multiple testing.

### Overlap with previous postpartum depression onset candidate genes

We compared our results to our previously reported candidate genes whose third trimester of pregnancy gene expression profiles were significantly associated with PPD-onset^22^. Of the 116 genes associated with PPD onset in our previous study, 84 genes were present in this study. From these, three genes (TMEM189, GALNT10 and FBXL20) were also associated with PPD onset in this study and not with depression per se (enrichment *P* = 0.08, indicating no significant overlap than expected by chance).

Next, we compared genes associated with PPD in this study with our previous study investigating gene expression and DNA methylation changes in a pharmacological model of depression where women were treated with the gonadotrophin-releasing hormone agonist (GnRHa) to induce depressive symptoms^24^. In the GnRHa study, we have previously shown that gene expression changes post-GnRHa induction directly relate oestrogen-induced biological changes with depressive symptoms and associated markers of serotonin-signalling in the brain, suggesting that individual variations in molecular sensitivity to oestrogen are associated with susceptibility to hormone-induced mood changes^23^.

A total of 7552 evaluated genes in this study overlapped with the GnRHa study. Of the genes associated with depressive symptoms (Table 2), change in depressive symptoms at 2 months postpartum (Table 3) or PPD onset (Table 4) at FDR *P* < 0.05, 10 (of 34, 29% overlap, *P* value = 4.2e-06), 17 (of 32, 53% overlap, *P* value = 2.1e-14) and 6 (of 17, 35% overlap, *P* = 1.2e-4) genes respectively were significantly associated with estradiol changes among the GnRHa-treated women.

Since the GnRhHa model in particular provoked biphasic changes in estradiol, these findings provide further evidence of the role of oestrogen signalling in PPD.

### Overlap with the previously reported list of core maternal genes

We investigated a list of 700 core maternal genes identified by Gammie and colleagues^32^ that showed significant gene expression changes across multiple brain regions. This list was synthesised via bioinformatics analysis using findings from four microarray studies of different maternal brain regions. Of the 700 maternal genes from Gammie et al.^32^, we had 455 in the current gene expression study that was detected above background and after quality control. Of these, 70/455 (15.4%) were significantly differentially expressed in one of the above analysis (Table 5) of association with depressive symptoms at 2 months postpartum or changes in depressive symptoms from recruitment to 2 months postpartum or differentially expressed between the PPD and the control groups. This is a significant enrichment than expected by chance (*P* = 7.9e-05).

**Table 5:** Comparison of differentially expressed genes at the third trimester of pregnancy associated with PPD with previously reported maternal genes by Gammie et al. 2016.

Symbol	Probe	Chr	*P* value_EPDSv2	FDR_EPDSv2	*P* value_diffEPDSv2	FDR_diffEPDSv2	*P* value_PPD	FDR_PPD	*P* value_Dep	FDR_Dep
ADCY5	111	3	0.014050053	0.554047576	0.002962235	0.216262025	0.91178575	0.99980319	0.98965523	0.99997367
ASAP2	8853	2	0.23315749	0.999797241	0.832816451	0.999245113	0.4734246	0.99980319	0.73193125	0.99997367
ATP10A	57194	15	0.071056678	0.999797241	0.041084579	0.660254558	0.99541483	0.99980319	0.94005609	0.99997367
BCL2L1	598	20	0.011365889	0.515005387	0.914054201	0.999245113	0.98761521	0.99980319	0.32804887	0.99997367
CADPS	8618	3	0.212269446	0.999797241	0.795011	0.999245113	0.77701682	0.99980319	0.81620633	0.99997367
CCDC85B	11007	11	0.025452704	0.739222531	0.363283011	0.999245113	0.05392936	0.99980319	0.26021851	0.99997367
CD200	4345	3	0.093184684	0.999797241	0.232688233	0.969329781	0.00999878	0.80474158	0.02667391	0.99997367
CNTNAP3	79937	9	0.016675728	0.616660406	0.024084768	0.559265472	0.71961007	0.99980319	0.3454232	0.99997367
DAAM2	23500	6	0.618068822	0.999797241	0.465553179	0.999245113	0.33148853	0.99980319	0.43262365	0.99997367
DDAH2	23564	6	0.121695981	0.999797241	0.633074639	0.999245113	0.10762088	0.99980319	0.34735339	0.99997367
DLC1	10395	8	0.18544046	0.999797241	0.020026641	0.514369946	0.19652235	0.99980319	0.42960279	0.99997367
DSCAM	1826	21	0.002077118	0.191346638	0.675666126	0.999245113	0.62745455	0.99980319	0.97833471	0.99997367
DZIP1	22873	13	0.268435651	0.999797241	0.567421782	0.999245113	0.70184475	0.99980319	0.95756208	0.99997367
ENG	2022	9	0.852264699	0.999797241	0.230231174	0.966439689	0.65484722	0.99980319	0.49902445	0.99997367
FBN1	2200	15	0.030794244	0.826999396	0.9026211	0.999245113	0.88818414	0.99980319	0.89595002	0.99997367
FLT1	2321	13	0.570727567	0.999797241	0.038153876	0.647066685	0.00242766	0.38734395	0.06800417	0.99997367
FRAS1	80144	4	0.008458146	0.439583873	0.39781099	0.999245113	0.95921892	0.99980319	0.93647918	0.99997367
FSTL5	56884	4	8.78E-05	0.023000406	0.050527822	0.708162007	0.00970811	0.8009674	0.03873353	0.99997367
FXYD1	5348	19	0.018867223	0.646818417	0.762825669	0.999245113	0.92048491	0.99980319	0.88225019	0.99997367
GCNT2	2651	6	0.212632395	0.999797241	0.024135941	0.559265472	0.91377176	0.99980319	0.72771845	0.99997367
GLDC	2731	9	0.018982052	0.646818417	0.039814013	0.653570948	0.2049579	0.99980319	0.36900048	0.99997367
GPR27	2850	NA	0.703341699	0.999797241	0.008356958	0.348306075	0.26369055	0.99980319	0.56382651	0.99997367
GPRIN3	285513	4	0.158689278	0.999797241	0.007280647	0.331404856	0.5722268	0.99980319	0.74894925	0.99997367
HIF1A	3091	14	0.083348331	0.999797241	0.805687086	0.999245113	0.00235364	0.38734395	0.05975394	0.99997367
HIST1H1C	3006	6	0.371095763	0.999797241	0.91982595	0.999245113	0.00481477	0.56509769	0.19892836	0.99997367
HIST1H2BK	85236	6	0.418176509	0.999797241	0.449859358	0.999245113	0.0580796	0.99980319	0.33328433	0.99997367
HMGB3	3149	X	0.001054142	0.116964982	0.170650959	0.957043264	0.57580736	0.99980319	0.49913663	0.99997367
HOMER1	9456	5	0.002422146	0.207713945	0.928286192	0.999245113	0.99781621	0.99980319	0.99212125	0.99997367
HPCAL4	51440	1	0.962836118	0.999797241	0.404980957	0.999245113	0.00948858	0.79615389	0.07530006	0.99997367
HSPB1	3315	7	0.774603671	0.999797241	0.008067963	0.341040786	0.02324166	0.99980319	0.24558202	0.99997367
LRRC29	26231	16	0.399050799	0.999797241	0.022297823	0.540070111	0.33786985	0.99980319	0.62596094	0.99997367
MAP3K15	389840	X	0.420717446	0.999797241	0.958895197	0.999245113	0.22671791	0.99980319	0.45515549	0.99997367
MCF2L	23263	13	0.398696592	0.999797241	0.001172201	0.13316409	0.25535639	0.99980319	0.20225951	0.99997367
MERTK	10461	2	0.101097195	0.999797241	0.044390268	0.678278078	0.63289989	0.99980319	0.40555607	0.99997367
NEURL1B	54492	5	0.036201122	0.879054327	0.11249574	0.877771504	0.94603704	0.99980319	0.9241486	0.99997367
NEXN	91624	1	0.388511732	0.999797241	0.021162427	0.524521154	0.3702592	0.99980319	0.6356162	0.99997367
NR1D1	9572	17	0.244295838	0.999797241	0.032187846	0.615427276	0.48714116	0.99980319	0.99394571	0.99997367
NR4A1	3164	12	0.54874689	0.999797241	0.811163817	0.999245113	0.03001189	0.99980319	0.08679762	0.99997367
NSUN3	63899	3	0.586484515	0.999797241	0.459638237	0.999245113	0.08934916	0.99980319	0.08254255	0.99997367
OLIG2	10215	21	0.063562425	0.999797241	0.026373718	0.585711441	0.00299288	0.42688463	0.0149956	0.99997367
PDP1	54704	8	0.885184882	0.999797241	0.669359061	0.999245113	0.64335095	0.99980319	0.58963618	0.99997367
PHLPP2	23035	16	0.755938138	0.999797241	0.596468698	0.999245113	0.03355505	0.99980319	0.09609877	0.99997367
PLCB1	23236	20	0.348381116	0.999797241	0.020000685	0.514369946	0.50119888	0.99980319	0.79923428	0.99997367
PLIN4	729359	19	0.408653941	0.999797241	0.033853456	0.62292633	0.48816799	0.99980319	0.35878095	0.99997367
PLLP	51090	16	0.746268876	0.999797241	0.006307858	0.305379969	0.75307827	0.99980319	0.66400787	0.99997367
PPP1R12B	4660	1	0.04338289	0.947889322	0.039379693	0.652530615	0.28069488	0.99980319	0.38859106	0.99997367
PSD	5662	10	0.923810949	0.999797241	0.042759347	0.672382742	0.6654271	0.99980319	0.45005221	0.99997367
PSPH	5723	7	0.686104071	0.999797241	0.873717886	0.999245113	0.42450925	0.99980319	0.92148676	0.99997367
RAB30	27314	11	0.71805606	0.999797241	0.061149329	0.745952208	0.01561673	0.94562271	0.06022857	0.99997367
RASA3	22821	13	0.772698055	0.999797241	0.011718405	0.413266918	0.68529277	0.99980319	0.88951742	0.99997367
RASGEF1B	153020	4	0.606014303	0.999797241	0.900764539	0.999245113	0.01995988	0.99980319	0.04431899	0.99997367
RFTN1	23180	3	0.509223491	0.999797241	0.001629844	0.158502285	0.566442	0.99980319	0.97046749	0.99997367
SBK1	388228	16	0.814900153	0.999797241	0.032447549	0.615427276	0.94079819	0.99980319	0.76509714	0.99997367
SERINC2	347735	1	0.001346019	0.142800404	0.098238466	0.848244582	0.93519291	0.99980319	0.3510916	0.99997367
SGSM1	129049	22	0.426624611	0.999797241	0.347460956	0.999245113	0.96549994	0.99980319	0.8219647	0.99997367
SLC2A1	6513	1	0.197794608	0.999797241	0.73633701	0.999245113	0.00110525	0.21827851	0.0049442	0.99997367
SLC38A5	92745	X	0.009995578	0.474076994	0.893153414	0.999245113	0.84453604	0.99980319	0.35205746	0.99997367
SLC4A4	8671	4	0.960039114	0.999797241	0.458677999	0.999245113	0.02456949	0.99980319	0.04855063	0.99997367
SLC4A8	9498	12	0.466064002	0.999797241	0.029660741	0.604610542	0.02353749	0.99980319	0.15735593	0.99997367
SMOX	54498	20	0.060547007	0.999797241	0.033388089	0.621163618	0.19395148	0.99980319	0.93632637	0.99997367
SNRK	54861	3	0.221630481	0.999797241	0.052251277	0.711293364	0.00027156	0.07416982	0.01404638	0.99997367
SOCS3	9021	17	0.902530531	0.999797241	0.090285457	0.832799778	0.97152061	0.99980319	0.99901805	0.99997367
SORCS2	57537	4	0.1797705	0.999797241	0.001092399	0.133018019	0.01452102	0.92830067	0.30719731	0.99997367
TMEM165	55858	4	0.990113374	0.999797241	0.323611169	0.999245113	0.69745403	0.99980319	0.67185592	0.99997367
TNFRSF12A	51330	16	0.69170039	0.999797241	0.213123462	0.964674548	0.68818696	0.99980319	0.83076288	0.99997367
TRIM9	114088	14	0.642086487	0.999797241	0.206442423	0.961442886	0.01238762	0.88646873	0.04950842	0.99997367
ZER1	10444	9	0.008606227	0.443784651	0.288011553	0.994394748	0.4064001	0.99980319	0.27713802	0.99997367
ZFP57	346171	6	0.688934236	0.999797241	9.57E-12	3.07E-08	0.00014379	0.04988752	0.0056805	0.99997367
ZNF512B	57473	20	0.188375011	0.999797241	0.005563967	0.293928586	0.93831436	0.99980319	0.56044337	0.99997367
ZXDB	158586	X	0.309677897	0.999797241	0.862922218	0.999245113	0.39484065	0.99980319	0.6766835	0.99997367

*EPDS* Edinburgh Postnatal Depression Scale symptoms at 2 months postpartum, *diffEPDS* difference in EPDS symptoms from the third trimester of pregnancy to 2 months postpartum, *PPD* EPDS symptoms in the PPD group, *Dep* EPDS symptoms in the depression group, *GnRHa_estradiol* genes associated with estradiol in the GnRHa study, *FDR* *P* values after false discovery rate multiple testing.

Among genes that showed significant maternal brain gene expression changes^32^ were interesting candidate genes including the ZFP57 gene, a maternal-zygotic effect gene involved in the stable maintenance of methylation imprints during development^33^ and the NR1D1 gene which has been highlighted as a key PPD gene and is linked to the steroid receptor.

## Discussion

PPD is a common disorder that is frequently underdiagnosed and can lead to severe long-term negative health outcomes of the mother, infant and the family hence early identification of women at high risk for PPD is essential to allow timely preventive intervention and treatment before the symptoms of PPD begin to manifest.

The aim of this study was to evaluate genome-wide gene expression profiles of women from their third trimester of pregnancy to identify transcriptomic changes associated with PPD. To the best of our knowledge, this is the largest PPD gene expression study to date (*n* = 137), assessing early gene expression profiles during late pregnancy and their association with PPD.

A total of 71 genes were significantly associated with depression scores at 2 months postpartum, after correction for multiple testing. The PPD related genes were enriched for immune-related gene ontology biological processes including humoral immune response and neutrophil-mediated immunity and were overrepresented in the neutrophil degranulation and innate immune system pathways, suggesting a role of immune-pathway genes in PPD. These findings suggest that an altered immunological profile during pregnancy might play a role in PPD vulnerability. There are several theories linking depression with immune responses including the Pathogen Host Defense (PATHOS-D) hypothesis that suggests that depressive symptoms are integral components of immune-mediated host defence against pathogens, having an underlying evolutionary significance^34^. It remains to be established whether and at what extent the immune abnormalities observed are pre-existing even before pregnancy and how these change across pregnancy, possibly contributing to the development of PPD; larger longitudinal studies will uncover the exact mechanisms.

The top genes associated with 2 months postpartum-depressive scores included TNFRSF17, GYPA, JCHAIN, SMIM1, TRPM8, SPATA17, HEMGN, PRRT4 and ADAMS15. The TNFRSF17 gene is a member of the TNF-receptor superfamily, specifically leading to NF-kappaB and MAPK8/JNK activation, with a likely role in cell survival and proliferation. In another study^35^, during the third trimester of pregnancy, the levels of TNFRSF17 gene expression significantly upregulated in women with PPD as compared to euthymic pregnant women and depressed patients (depressed during pregnancy and postpartum). TNFRSF17 was also one of the immune response genes upregulated in patients with bipolar disorder^36^.

Next, we tested whether gene expression in the third trimester of pregnancy was associated with a difference in the third trimester of pregnancy to 2 months postpartum-depressive symptoms, as this was more likely to be of clinical relevance. We identified 66 genes whose gene expression profiles were associated with changes in depressive symptoms at a 5% FDR, these were substantially overlapped (50% at *P* < 0.05 and 21% at FDR 5%) with genes associated with only 2 months postpartum-depressive symptoms. The genes included OLFM4, RASAL2, BHZ, DEFA1B, DEFA4, C4BPA, CPA5, GPRC5C, LTF, ADAMTS15, CLEC12A and ELANE. The C4BPA has been reported in studies of individuals with psychotic experiences^37^ and schizophrenia^38^. The ELANE gene encodes for a protein that is found in neutrophils and plays a role in inflammation and in fighting infection.

When comparing the postpartum-onset group to euthymic controls (and using the depression group as a negative control), the study revealed 30 PPD-onset specific gene expression. Of the 30 genes, 16 genes were also significant in the difference in depressive symptoms (*P* < 0.05), 15 were also associated with depressive symptoms at 2 months postpartum and 8 of the genes were associated with both. Genes included MMP8, a matrix metalloproteinase–collagen degradation gene, previously reported to be associated with MDD in a genome-wide association study^39^. Other genes included SLC35F1, which was one of the significant genes for extraversion and neuroticism, and symptoms of anxiety, depression and psychological distress in a genome-wide association uncovering shared genetic effects among personality traits and mood states^40^.

Our previous research has investigated the transcriptomic landscape among women with PPD and identified that oestrogen signalling plays a key role in vulnerability to PPD. For instance, we and others had identified a panel of 116 genes significantly differentially expressed between women with PPD and euthymic controls^22^. These genes could predict with 88% accuracy which women went on to develop PPD within the sample and were enriched for those belonging to the oestrogen signalling pathway. In this study, we found three of these predictive PPD genes (TMEM189, GALNT10 and FBXL20) were also associated with PPD onset in the current study and not with depression per se. This overlap is consistent with chance alone (*P* = 0.80). The lack of enrichment in gene associations across the two studies could be the result of different samples and different technologies (microarray versus next-generation sequencing methods). Nevertheless, when comparing the gene expression profiles in PPD identified in this study with our previous pharmacological model of depression via GnRHa^24^, we found evidence that 29–53% of the genes were also associated with estradiol changes among the GnRHa-treated women (*P* values range = 1.2e-4–2.1e-14). Therefore, these results again emphasise the role of oestrogen signalling in PPD.

To further extrapolate and understand the function of the differentially expressed genes associated with PPD, the comparison was performed with the maternal genes identified by Gammie et al.^32^. This included a list of 700 core maternal genes that showed significant gene expression changes across multiple brain regions. Of the 455 genes present in the current gene expression study, 15.4% were significantly associated with PPD (enrichment *P* = 7.9e-05). Among genes that showed significant maternal brain gene expression changes^32^ was the ZFP57 gene, a maternal-zygotic effect gene that contributes towards the stable maintenance of methylation imprints during development^33^. This gene was significantly associated with depressive symptoms at 2 months postpartum in both discovery and replication cohorts, changes in depressive symptoms from late pregnancy to 2 months postpartum and was differentially expressed between the PPD and the control groups. Another interesting gene from the Gammie et al. study was the NR1D1 gene which was highlighted by the authors as a key PPD gene and is linked to the steroid receptor. NR1D1 gene expression is affected by estradiol, glucocorticoids and progesterone treatment, making this a key maternal brain gene, whose expression can be moderated by placenta-produced hormones and changes in these hormones. In this study, the NR1D1 gene was associated with depressive symptoms at 2 months postpartum.

This study has several limitations. Since this was a cohort-based study, some women (18%) were on medications and this likely influenced gene expression profiles, however, we did correct for medication status in the analysis. Given that only 11% of the women had PPD, to overcome the consequential issue of low power, we analysed both quantitative depression symptoms as well as PPD onset to better understand the influence of depressive symptoms on the transcriptome. Strengths of the study include that this is the largest gene expression study in PPD to date and we validate our results in other independent samples and show enrichment of the identified PPD genes in relevant studies.

In conclusion, this study interrogated the transcriptomic landscape during pregnancy and tested its association with PPD revealing novel genes and pathways associated with PPD and validating previous PPD candidates. Consistent with previous studies including our own research, this study strengthened the findings of altered immune genes in PPD and the role of oestrogen in the pathogenesis of PPD, suggesting avenues for treatment of PPD. These findings add to the existing knowledge of the molecular mechanisms and pathways associated with PPD, providing avenues for improved diagnosis and treatment.

## Supplementary information

Supplementary Table 1

## References

[1] O’Hara MW, Wisner KL (2014). Perinatal mental illness: definition, description and aetiology. Best. Pr. Res Clin. Obstet. Gynaecol..

[2] Sockol LE, Epperson CN, Barber JP (2013). Preventing postpartum depression: a meta-analytic review. Clin. Psychol. Rev..

[3] American Psychiatric Association. *Diagnostic and Statistical Manual of Mental Disorders*, 5th edn. (American Psychiatric Association, 2013.

[4] Lapato DM (2019). DNA methylation associated with postpartum depressive symptoms overlaps findings from a genome-wide association meta-analysis of depression. Clin. Epigenet..

[5] Putnam KT (2017). Clinical phenotypes of perinatal depression and time of symptom onset: analysis of data from an international consortium. Lancet Psychiatry.

[6] Ban L, Gibson JE, West J, Tata LJ (2010). Association between perinatal depression in mothers and the risk of childhood infections in offspring: a population-based cohort study. BMC Public Health.

[7] Gump BB (2009). Trajectories of maternal depressive symptoms over her child’s life span: relation to adrenocortical, cardiovascular, and emotional functioning in children. Dev. Psychopathol..

[8] Rahman A (2004). Mothers’ mental health and infant growth: a case-control study from Rawalpindi, Pakistan. Child Care Health Dev..

[9] Malm H (2016). Gestational exposure to selective serotonin reuptake inhibitors and offspring psychiatric disorders: a national register-based study. J. Am. Acad. Child Adolesc. Psychiatry.

[10] Rai D (2013). Parental depression, maternal antidepressant use during pregnancy, and risk of autism spectrum disorders: population based case-control study. BMJ.

[11] Stein A (2014). Effects of perinatal mental disorders on the fetus and child. Lancet.

[12] Sit D (2015). Suicidal ideation in depressed postpartum women: associations with childhood trauma, sleep disturbance and anxiety. J. Psychiatr. Res..

[13] Paulson JF, Bazemore SD (2010). Prenatal and postpartum depression in fathers and its association with maternal depression: a meta-analysis. J. Am. Med. Assoc..

[14] Austin MP (2004). Antenatal screening and early intervention for “perinatal” distress, depression and anxiety: where to from here?. Arch. Women’s Ment. Health.

[15] O’Connor E (2016). Primary care screening for and treatment of depression in pregnant and postpartum women: evidence report and systematic review for the US preventive services task force. J. Am. Med. Assoc..

[16] Guintivano J, Manuck T, Meltzer-Brody S (2018). Predictors of postpartum depression: a comprehensive review of the last decade of evidence. Clin. Obstet. Gynecol..

[17] Guintivano J (2018). Adverse life events, psychiatric history, and biological predictors of postpartum depression in an ethnically diverse sample of postpartum women. Psychol. Med..

[18] Leigh B, Milgrom J (2008). Risk factors for antenatal depression, postnatal depression and parenting stress. BMC Psychiatry.

[19] Mehta D (2012). The 5-HTTLPR polymorphism modulates the influence on environmental stressors on peripartum depression symptoms. J. Affect Disord..

[20] Doornbos B (2009). The development of peripartum depressive symptoms is associated with gene polymorphisms of MAOA, 5-HTT and COMT. Prog. Neuropsychopharmacol. Biol. Psychiatry.

[21] Figueira P (2010). An association study between the Val66Met polymorphism of the BDNF gene and postpartum depression. Arch. Women’s Ment. Health.

[22] Mehta D (2014). Early predictive biomarkers for postpartum depression point to a role for estrogen receptor signaling. Psychol. Med..

[23] Mehta D (2019). Evidence for oestrogen sensitivity in perinatal depression: pharmacological sex hormone manipulation study. Br. J. Psychiatry.

[24] Mehta D (2019). Genome-wide gene expression in a pharmacological hormonal transition model and its relation to depressive symptoms. Acta Psychiatr. Scand..

[25] Stuebe AM (2019). The mood, mother, and infant study: associations between maternal mood in pregnancy and breastfeeding outcome. Breastfeed. Med..

[26] Cox, J. & Holden, J. *Perinatal Mental Health: A Guide to the Edinburgh Postnatal Depression Scale (EPDS)* (Royal College of Psychiatrists, 2003).

[27] Pearson RM (2013). Maternal depression during pregnancy and the postnatal period: risks and possible mechanisms for offspring depression at age 18 years. JAMA Psychiatry.

[28] Lee HH, Kim TH (2014). Screening depression during and after pregnancy using the EPDS. Arch. Gynecol. Obstet..

[29] Meijer JL (2014). Predictive accuracy of Edinburgh postnatal depression scale assessment during pregnancy for the risk of developing postpartum depressive symptoms: a prospective cohort study. BJOG.

[30] Robinson MD, McCarthy DJ, Smyth GK (2010). edgeR: a Bioconductor package for differential expression analysis of digital gene expression data. Bioinformatics.

[31] Liao Y (2019). WebGestalt 2019: gene set analysis toolkit with revamped UIs and APIs. Nucleic Acids Res..

[32] Gammie SC (2016). Genetic and neuroendocrine regulation of the postpartum brain. Front. Neuroendocrinol..

[33] Li X (2008). A maternal-zygotic effect gene, Zfp57, maintains both maternal and paternal imprints. Dev. Cell..

[34] Raison CL, Miller AH (2013). The evolutionary significance of depression in Pathogen Host Defense (PATHOS-D). Mol. Psychiatry.

[35] Petralia MC (2019). Retrospective follow-up analysis of the transcriptomic patterns of cytokines, cytokine receptors and chemokines at preconception and during pregnancy, in women with post-partum depression. Exp. Ther. Med..

[36] Ryan MM (2006). Gene expression analysis of bipolar disorder reveals downregulation of the ubiquitin cycle and alterations in synaptic genes. Mol. Psychiatry.

[37] Focking M (2019). Complement pathway changes at age 12 are associated with psychotic experiences at age 18 in a longitudinal population-based study: evidence for a role of stress. Mol. Psychiatry.

[38] Wang S (2015). An evaluation of association between common variants in C4BPB/C4BPA genes and schizophrenia. Neurosci. Lett..

[39] Song GG, Kim JH, Lee YH (2013). Genome-wide pathway analysis in major depressive disorder. J. Mol. Neurosci..

[40] Luciano M (2012). Genome-wide association uncovers shared genetic effects among personality traits and mood states. Am. J. Med. Genet. B Neuropsychiatr. Genet..

